# Evaluation of Platelet Indices and Inflammation Markers in Preeclampsia

**DOI:** 10.3390/jcm14051406

**Published:** 2025-02-20

**Authors:** Betül Tokgöz Çakır, Gizem Aktemur, Gülşan Karabay, Zeynep Şeyhanlı, Sevinç Çetin, Ahmet Arif Filiz, Nazan Vanlı Tonyalı, Ali Turhan Çağlar

**Affiliations:** 1 Department of Perinatology, Ankara Etlik City Hospital, Ankara 06170, Türkiye; drgizemkizilbuga@gmail.com (G.A.); drgulsankarabay@gmail.com (G.K.); drzeynepseyhanli@gmail.com (Z.Ş.); ahmetarif_filiz@hotmail.com (A.A.F.); nazanvanli@saglik.gov.tr (N.V.T.); turhan_caglar@yahoo.com (A.T.Ç.); 2 Department of Obstetrics and Gynecology, Ankara Etlik City Hospital, Ankara 06170, Türkiye; sevinc.cetin@hotmail.com

**Keywords:** inflammation markers, platelet indices, platelet distribution width (PDW)

## Abstract

**Background**: Preeclampsia is a serious pregnancy complication known to be related to the pathophysiology of platelet dysfunction and inflammation. The aim of this study was to investigate the role of platelet indices and inflammatory markers in preeclampsia and their importance in predicting adverse neonatal outcomes. **Methods**: A total of 118 preeclampsia cases (84 with mild preeclampsia and 34 with severe preeclampsia) and 118 healthy pregnant women were included in the study. Blood samples obtained at the time of preeclampsia diagnosis were analyzed for platelet indices (platelet count (PC), platelet distribution width (PDW), mean platelet volume (MPV), and platelet/large cell ratio (P-LCR)) and inflammation indices (neutrophil-to-lymphocyte ratio (NLR), monocyte-to-lymphocyte ratio (MLR), platelet-to-lymphocyte ratio (PLR), and mean platelet volume-to-lymphocyte ratio (MPVLR)). **Results**: The PC and PLR were lower in the severe preeclampsia group compared to the other groups. The PDW was higher in both mild and severe preeclampsia groups compared to the control group. A PDW value above 13.15 was identified as a significant predictor of composite adverse neonatal outcomes (area under the curve (AUC): 0.633; sensitivity: 60.9%; specificity: 58%). **Conclusions**: PC and PLR decrease in severe preeclampsia. This study highlights the potential of PDW as a marker for predicting adverse neonatal outcomes in preeclampsia.

## 1. Introduction

Preeclampsia is defined as hypertension and proteinuria or new-onset preeclampsia-related findings without proteinuria after 20 weeks of gestation in a pregnant woman previously known to be normotensive [1]. Preeclampsia is a leading cause of maternal and perinatal mortality, particularly in low- and middle-income countries where access to adequate prenatal care may be limited, affecting approximately four percent of pregnancies worldwide [2]. Placental ischemia, oxidative stress, and the insufficient remodeling of spiral arteries lead to endothelial injury [3]. This is followed by coagulation and platelet activation and increased extracellular vesicle formation, and a new thromboinflammatory process begins at the maternal–embryonic interface [4]. In addition to genetic, immunological, and environmental factors, inflammation is also known to play an important role in the pathogenesis of preeclampsia [5]. Furthermore, research indicates that the High-Temperature Requirement Protease A protein, linked to inflammatory diseases, significantly contributes to the pathogenesis of preeclampsia, highlighting that this condition is marked by inflammation [6]. This inflammatory state and associated thromboinflammatory mechanisms are reflected in various hematological parameters, which are both accessible and informative in clinical practice. These indices include platelet count (PC), mean platelet volume (MPV), platelet distribution width (PDW), and plateletcrit (PCT) [7]. Among these, PDW, an indicator of platelet size heterogeneity, is a marker of platelet function [8]. It is being evaluated as a potential indicator for the risk prediction of thromboinflammatory conditions, being particularly associated with P-selectin-dependent platelet activation [9].

Furthermore, the neutrophil-to-lymphocyte ratio (NLR), monocyte-to-lymphocyte ratio (MLR), and platelet-to-lymphocyte ratio (PLR) are systemic inflammatory markers derived from routine blood tests [10]. There are many studies suggesting that these simple and cost-effective markers are valuable in the evaluation of pregnancy morbidities and prognoses [11,12,13]. Although platelet indices and inflammatory markers have been investigated for their role in predicting the onset and severity of preeclampsia, data on their relationship with adverse neonatal outcomes remain limited [14,15,16].

The timing of delivery in preeclamptic women should be carefully considered to ensure optimal outcomes for both mother and baby, especially between 34 and 37 weeks, and this should be assessed on an individual basis [17]. However, there is insufficient and inconclusive evidence in the literature to reliably predict both maternal and fetal outcomes or adverse obstetric outcomes in cases of preeclampsia. Clinicians must consider both the complications associated with prematurity and maternal risks. Considering that inflammation and platelet activation play a role in the pathophysiology, we aimed to investigate maternal blood parameters and platelet indices in pregnant women diagnosed with preeclampsia. Secondarily, we aimed to evaluate the relationship of these tests with adverse neonatal outcomes.

## 2. Materials and Methods

This retrospective study included patients who were followed up with a diagnosis of preeclampsia at Ankara Etlik City Hospital Perinatology Clinic (Ankara, Turkey) between January 2023 and November 2024. Ethical approval was obtained from the hospital’s ethics committee for the study (approval number: AESH-BADEK-2024-1141; date: 25 December 2024). Preeclampsia was defined as the onset of new hypertension after 20 weeks of gestation (systolic blood pressure (SBP) > 140 mmHg or diastolic blood pressure (DBP) > 90 mmHg, confirmed by two separate measurements at least six hours apart) and significant proteinuria, or as hypertension accompanied by evidence of organ dysfunction, including thrombocytopenia, impaired liver function, renal insufficiency, pulmonary edema, or new-onset cerebral or visual disturbances [18].

A total of 118 pregnant women diagnosed with preeclampsia were selected as the patient group, while 118 normotensive pregnant women with similar age and body mass index (BMI) were selected as the control group. Then, subgroup analysis was performed for preeclampsia, including 84 mild preeclampsia cases, 34 severe preeclampsia cases, and 118 healthy pregnant women. The severity of preeclampsia was assessed based on SBP ≥ 160 mm Hg, DBP ≥ 110 mm Hg, or the manifestation of particular clinical indicators, such as renal insufficiency, pulmonary edema, microvascular disease, thrombocytopenia, compromised liver function, and significant peripheral organ involvement (visual disturbances and headaches). Mild preeclampsia was diagnosed when patients fulfilled the criteria for preeclampsia but did not meet the criteria for severe preeclampsia [18,19].

Pregnant women between the ages of 17 and 45 and without any known chronic disease were included in the study. Patients who had previously used aspirin and/or used any medication other than multivitamins during pregnancy were excluded from the study as these medications may affect platelet function. The complete blood parameters of the patients were examined in the week when preeclampsia was diagnosed, which, according to patient records, occurred between 24 and 39 weeks of gestation. Blood samples were collected using standard venipuncture techniques and analyzed within two hours of collection to ensure accuracy. Platelet indices (PDW, MPV, and platelet/large cell ratio (P-LCR)) and other parameters (hemoglobin, white blood cells (WBCs), lymphocytes, neutrophils, and monocytes) were measured using an automated hematology analyzer (Sysmex XN 1000, Sysmex Corporation, Kobe, Japan), which employs impedance and optical fluorescence technology for complete blood count and differential leukocyte analysis. The analyzer was calibrated periodically according to the manufacturer’s guidelines, and internal quality control samples were run daily to ensure measurement reliability. Their values, including hemoglobin, WBCs, lymphocytes, neutrophils, monocytes, platelets, PCT, and PDW, were recorded. In addition, the NLR, MLR, monocyte-to-platelet ratio, and mean platelet volume-to-lymphocyte ratio (MPVLR) were calculated. Significant differences between the groups were determined.

Delivery data and outcomes were obtained from the hospital’s electronic record system. As a secondary outcome, we examined whether these parameters could predict adverse neonatal outcomes in the groups. Composite adverse neonatal outcomes (CANOs) were defined as one or more of the following events: intrauterine fetal death, need for admission to the neonatal intensive care unit (NICU), 5 min APGAR score (which assesses Appearance (skin color), Pulse (heart rate), Grimace (reflex irritability), Activity (muscle tone), and Respiration (breathing effort)) < 7, or umbilical cord blood pH < 7.

### Statistical Analysis

The statistical analysis was performed using SPSS version 29.0. Continuous variables were tested for normality using the Shapiro–Wilk test. Data are presented as mean ± standard deviation (SD) for normally distributed variables and median (Q1–Q3) for non-normally distributed variables. Categorical variables are expressed as frequencies and percentages. For comparisons among the three groups (mild preeclampsia, severe preeclampsia, and control), a one-way ANOVA or the Kruskal–Wallis test was used for continuous variables, depending on the normality of the data. Post hoc pairwise comparisons were conducted using the Tukey test for parametric data or Dunn’s test with Bonferroni correction for non-parametric data. The chi-square test or Fisher’s exact test was applied for categorical variables as appropriate. Receiver operating characteristic (ROC) curve analysis was used to evaluate the discriminatory power of the platelet indices (PDW, PLR, and MLR) for predicting CANOs. The area under the curve (AUC) with 95% confidence intervals (95% CIs) was calculated, and the optimal cut-off values were determined using the Youden index. Sensitivity and specificity were also reported. A *p*-value < 0.05 was considered statistically significant.

## 3. Results

The demographic characteristics of the study population are shown in Table 1. There were no significant differences between the mild preeclampsia, severe preeclampsia, and control groups with respect to maternal age, BMI, gravidity, parity, primiparity, or smoking habits (*p* > 0.05).

The laboratory data of the patients are summarized in Table 2. The hemoglobin levels were significantly higher in the group with mild preeclampsia than in the control group (*p* = 0.016). The PC was lower in the severe preeclampsia group than in the control group (*p* = 0.007) and the mild preeclampsia group (*p* = 0.024). The PDW values (%) were significantly higher in the severe preeclampsia group than in the control group (*p* < 0.001) and in the mild preeclampsia group (*p* = 0.001). The PLR values were lower in the severe preeclampsia group than in the control group (*p* = 0.011). In addition, the MLR values were significantly lower in the severe preeclampsia group compared to the control group (*p* = 0.036). No statistically significant difference was found between the groups for other hematologic and biochemical parameters. The amount of proteinuria was significantly higher in the severe preeclampsia group than in the mild preeclampsia group (*p* = 0.009).

The neonatal outcomes were compared between the groups, as shown in Table 3. The mild and severe preeclampsia groups exhibited significant differences in birth weight, preterm delivery, cesarean section rate, APGAR scores, NICU admission, and CANO rate compared to the control group. In the mild preeclampsia group, the birth week was earlier (*p* < 0.001), the birth weight lower (*p* < 0.001), and the preterm birth rate higher than in the control group (*p* < 0.001). In the severe preeclampsia group, the gestational age was not significantly different from that in the mild preeclampsia group (*p* = 0.153), but the birth weight was lower (*p* < 0.001), and the preterm birth rate was significantly higher (*p* < 0.001). The rate of admission to the NICU was higher in the severe preeclampsia group than in the mild preeclampsia group (*p* < 0.001), and the APGAR scores for the first and fifth minute were significantly lower (*p* = 0.005 and *p* = 0.003). The CANO rates were also significantly higher in the severe preeclampsia group than in the mild preeclampsia group (*p* = 0.004).

**Figure 1:**
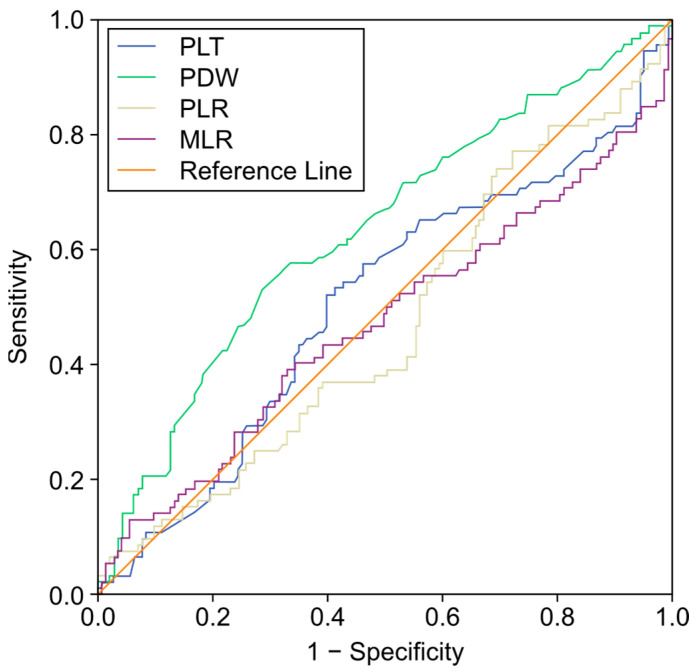
Receiver operating characteristic (ROC) analysis for platelet (PLT), platelet distribution width (PDW), platelet-to-lymphocyte ratio (PLR), and monocyte-to-lymphocyte ratio (MLR) in predicting composite adverse neonatal outcomes (CANOs).

Figure 1 shows the ROC analysis for the discriminatory power of platelets, PDW, PLR, and MLR for CANOs. The ROC analysis showed that PDW had the highest discriminatory power among the variables analyzed (AUC: 0.633; 95% CI: 0.559–0.707; *p* = 0.001). The optimal cut-off point for PDW was calculated to be 13.15, at which a sensitivity of 60.9% and a specificity of 58.0% were found. On the other hand, the variables platelet (PLT), PLR, and MLR (AUC values of 0.507, 0.471, and 0.472, respectively) were found to have no significant discriminatory power.

## 4. Discussion

Preeclampsia is a serious obstetric complication that affects endothelial dysfunction and the blood coagulation system. In this study, we investigated inflammatory markers and platelet indices by dividing preeclampsia into the subgroups of mild and severe preeclampsia. The PC and PLR were lower in the severe preeclampsia group compared to the other groups. Additionally, our results showed that CANOs can be predicted in preeclampsia, especially with PDW values above 13.15 (AUC: 0.633; sensitivity: 60.9%; specificity: 58%). We believe that the findings from this study can help in informing practice in the follow-up of preeclampsia and in determining the need for extended intensive care for the newborn.

Platelet indices are derived from the complete blood count. The indices encompass the MPV and the PDW, indicative of platelet size distribution; the PC, representing the volume of platelets per 100 mL of total blood; and the P-LCR, which denotes the percentage of platelets exceeding 12 fL in size. The data in the literature on platelet indices in pregnant women with preeclampsia are contradictory. It has been found that a low PC and a high MPV are associated with the development of preeclampsia [20,21]. One study found that a low PC and a high PDW value were associated with preeclampsia [22]. In our study, we found that the PC decreased significantly in severe preeclampsia, whereas there was no significant difference in mild preeclampsia. We selected normotensive pregnant women of similar age and BMI for the control group, and excluded patients who used any medication (including aspirin) other than multivitamins during pregnancy because of their possible effects on platelet functions. Because we kept these variables affecting PC similar, we hypothesize that PC was different only in severe preeclampsia, in which the peripheral organs are severely affected. Consistent with the literature, the PDW was higher in patients with both severe and mild preeclampsia. This could be due to increased platelet activation during preeclampsia and an increase in platelet heterogeneity in the circulation. It is also noteworthy that PDW was associated with poor obstetric outcomes in our study. It was revealed that PDW had the highest discriminatory power among the analyzed variables, but no significant change in MPV was observed. This difference could be due to the sample size of the study, population characteristics, or different clinical phenotypes of preeclampsia. Although MPV is used as an indirect indicator of endothelial dysfunction and platelet activation in preeclampsia [23], our results suggest that the diagnostic value of this parameter may not be consistent.

The possible pathophysiological mechanisms of preeclampsia include placental hypoperfusion, endothelial dysfunction, oxidative stress, inflammation, and immune abnormalities [14]. Systemic inflammatory indicators, such as NLR, PLR, and MLR, derived from peripheral blood cells have recently gained importance due to their ease of measurement and accessibility [24]. These markers have been considered indicators for forecasting the presence and severity of preeclampsia; however, inconsistent findings have been reported thus far. Studies have shown that NLR is either significantly higher or does not change significantly in preeclampsia. The research conducted by Wang et al. demonstrated that NLR and MLR possess good diagnostic accuracy in differentiating pregnant women with preeclampsia from healthy controls [25]. NLR has been identified as a reliable marker for predicting the severity of preeclampsia (AUC: 0.71; sensitivity: 53%; and specificity: 83%). Nonetheless, the study did not specify the gestational weeks during which blood samples were collected, nor were the patients’ medication status or aspirin usage included in the exclusion criteria. We made sure that the number of gestational weeks were the same in both the study and control groups, and that patients using any medication other than routine prenatal multivitamins were excluded. Additionally, a meta-analysis indicated a pooled mean difference of 1.44 in NLR between preeclampsia and healthy controls [26]. This finding indicates that NLR may be linked to the pathophysiology of preeclampsia and could serve as a possible diagnostic marker for the condition. It is important to acknowledge that meta-analyses are typically influenced by the methodological heterogeneity of many studies. Therefore, in our study, we aimed to obtain consistent results with a more homogeneous control group. In the study by Yavuzcan et al., it was suggested that there was no significant difference between women with severe preeclampsia and healthy pregnant women in terms of NLR, PLR, and MPV levels [27]. Similarly, in the study by Yücel et al. including 210 pregnant women, it was reported that there was no difference between the preeclampsia group and the control group in terms of NLR levels [15]. The outcomes of our investigation corroborate these conclusions. Our results suggest that the inflammatory response may vary from person to person and that NLR may not be a consistent biomarker in preeclampsia subgroups. In addition, study population characteristics, sample size, and other modulators of inflammation may be among the factors that may explain this difference. Additionally, the timing of blood sampling may have played a role in this discrepancy.

A meta-analysis found that PLR gradually decreased as preeclampsia progressed during the last two trimesters, most likely due to the increased systemic inflammation that preeclampsia causes [28]. Consistent with the literature, our findings indicate a reduction in PLR, particularly in severe preeclampsia; however, the PLR values in preeclampsia without severe characteristics were comparable to those in the control groups. It is reasonable for PLR to be lower in severe preeclampsia, where peripheral organ damage is known to be prominent.

In our study, the MLR was found to be low in the preeclampsia group. However, in addition to studies in the literature reporting that MLR increases or does not change in the case of preeclampsia, there is also a study indicating that MLR was lower in preeclamptic patients in the 18–25 age group and that there was no significant difference in preeclamptic women in the 26–35 age group [25,29,30]. These inconsistent results suggest that the MLR may not be a reliable marker for the diagnosis and follow-up of preeclampsia. The MLR is considered an inflammatory marker associated with an increase in monocyte count and/or a decrease in lymphocyte count. However, the decreased MLR seen in our study suggests that preeclampsia may result from various pathophysiological mechanisms. The lack of a significant increase in lymphocyte count in severe preeclampsia, along with potential mechanisms such as monocyte apoptosis, may explain this finding. Additionally, our study population was recruited from a single center, which could reflect specific demographic, genetic, or environmental factors influencing the inflammatory profile. The heterogeneous nature of preeclampsia pathogenesis may contribute to the variability in clinical presentations and inflammatory responses among different patient populations.

Predicting adverse neonatal outcomes and facilitating prompt transfer to an NICU is crucial for clinicians, as early intervention can greatly influence neonatal morbidity and mortality. Preeclampsia, a multifaceted multisystem condition, is known to elevate the risk of adverse neonatal outcomes due to its correlation with uteroplacental insufficiency, intrauterine growth restriction, and preterm birth [31]. Consistent with previous literature [32], our study found that neonates born to mothers with preeclampsia had higher rates of preterm delivery, lower APGAR scores, and an increased need for NICU admission compared to those in the control group. These findings emphasize the significant impact of preeclampsia on newborn health and reinforce the necessity of diligent prenatal monitoring and early risk assessment.

The primary limitation of our study is its retrospective design. Although the use of data from a single laboratory increases the reliability of the results by ensuring the uniformity of the measurement methods, the fact that the study was conducted in a single tertiary care institution may introduce biases related to local habits, demographic characteristics, or specific laboratory techniques. Furthermore, although our analysis excluded aspirin users and individuals with chronic diseases to reduce confounding variables, it is possible that undiagnosed subclinical infections or unreported drug use may have affected the platelet indices. Concomitant inflammatory conditions may potentially affect platelet function. Moreover, although PDW was significant in predicting CANOs, its sensitivity and specificity were relatively limited. Future multifactorial modeling and prediction studies may increase the reliability of the results. Future multicenter studies with larger samples are needed to confirm and extend the results of this study. Additionally, future research could focus on exploring other potential biomarkers and combining various inflammatory markers to improve predictive accuracy. Longitudinal studies assessing changes in platelet indices over time may also provide valuable insights into the pathophysiology of adverse neonatal outcomes.

## 5. Conclusions

This study emphasizes that PDW and inflammatory markers, such as the PLR, offer significant insights into the pathophysiology of preeclampsia. PDW has emerged as a potential predictor of adverse neonatal outcomes, but its sensitivity and specificity are relatively limited. Future modeling studies incorporating PDW may increase its predictive power. The PC and PLR were markedly decreased in severe preeclampsia, indicating their potential usefulness in evaluating illness severity. Employing accessible blood markers may improve risk classification and clinical management in preeclampsia. Given the multifactorial nature of adverse neonatal outcomes in preeclampsia, future studies should explore the combined predictive value of PDW with other hematological, biochemical, and Doppler ultrasound parameters to improve risk stratification and clinical decision making. Additionally, larger multicenter studies are needed to validate our findings and determine whether PDW can be incorporated into routine clinical practice as a reliable prognostic marker.

## Figures and Tables

**Table 1:** Pregnancy characteristics of the study population.

	Mild Preeclampsia (*n* = 84)	Severe Preeclampsia (*n* = 34)	Control (*n* = 118)	*p*-Value
Maternal age (years), mean ± SD	30.68 ± 6.36	29.22 ± 5.71	29.22 ± 4.9	0.178
BMI (kg/m^2^), mean ± SD	34.41 ± 6.56	32.71 ± 5.83	32.70 ± 4.22	0.068
Gravidity median, Q1–Q3	2 (1–3)	2 (1–3)	2 (1–3)	0.679
Parity median, Q1–Q3	1 (0–2)	0.5 (0–1)	1 (0–1)	0.560
Primiparity (n, %)	40 (47.6%)	18 (52.9%)	57 (48.3%)	0.864
Smoker (n, %)	7 (8.3%)	3 (8.8%)	4 (3.4%)	0.254
Gestational age at sampling median, Q1–Q3	34 (30–36)	32 (31–35)	33 (30–35)	0.798

BMI: body mass index; SD: standard deviation.

**Table 2:** Comparison of platelet indices and inflammation markers in mild and severe preeclampsia and control groups.

	Mild Preeclampsia (n = 84)	Severe Preeclampsia (n = 34)	Control (n = 118)	*p*-Value	Post Hoc Comparisons *p*-Value		
					Mild–Severe	Mild–Control	Severe–Control
Hemoglobin, g/dL	12.00 ± 1.28	11.99 ± 1.35	11.54 ± 1.31	**0.032**	0.989	**0.016**	0.080
WBC (×10⁹/L)	10.84 (9.34–12.74)	11.13 (8.79–15.34)	10.45 (8.56–12.00)	0.132			
Neutrophil (×10^9^/L)	8.21 (6.65–9.92)	8.60 (5.93–11.51)	7.93 (6.61–9.28)	0.612			
Platelet (×10^9^/L)	245.57 ± 60.22	215.14 ± 75.80	249.75 ± 66.36	**0.025**	**0.024**	0.655	**0.007**
Lymphocyte (×10^9^/L)	2.02 (1.52–2.36)	2.00 (1.66–2.19)	1.93 (1.64–2.18)	0.917			
Monocyte (×10^9^/L)	0.68 (0.49–0.85)	0.60 (0.38–0.81)	0.71 (0.61–0.89)	0.055			
MPV (×10^9^/L)	10.40 (10.15–11.55)	11.20 (10.30–12.10)	10.50 (10.10–11.50)	0.104			
PCT (%)	0.27 (0.23–0.31)	0.26 (0.20–0.30)	0.25 (0.22–0.30)	0.272			
PLCR (%)	31.46 (24.60–37.39)	35.80 (29.10–40.40)	31.86 (26.20–37.37)	0.084			
PDW (%)	14.10 (11.60–16.40)	15.00 (12.60–18.20)	12.15 (10.70–14.20)	**<0.001**	0.502	**0.001**	**<0.001**
NLR	4.22 (3.10–6.07)	4.04 (2.91–6.70)	4.03 (3.19–5.40)	0.887			
PLR	124 (102–160)	101 (79–121)	128 (101–163)	**0.010**	**0.020**	0.990	**0.011**
MLR	0.34 (0.25–0.45)	0.32 (0.21–0.42)	0.37 (0.31–0.48)	**0.016**	0.999	0.105	**0.036**
MPVLR	5.26 (4.28–7.31)	5.70 (6.63–7.89)	5.42 (4.71–6.89)	0.776			
Proteinuria	501 (384–958)	968 (492–3759)	NA	**0.009**			

Abbreviations: WBC: white blood cell; MPV: mean platelet volume; PCT: plateletcrit; PLCR: platelet/large cell ratio; PDW: platelet distribution width; NLR: neutrophil-to-lymphocyte ratio; PLR: platelet-to-lymphocyte ratio; MLR: monocyte-to-lymphocyte ratio; MPVLR: mean platelet volume-to-lymphocyte ratio. NA: Not Available.

**Table 3:** Neonatal outcomes in mild and severe preeclampsia compared to control groups.

	Mild Preeclampsia (n = 84)	Severe Preeclampsia (n = 34)	Control (n = 118)	*p*-Value	Post Hoc Comparisons *p*-Value		
					Mild–Severe	Mild–Control	Severe–Control
Gestational age at delivery (week)	37 (35–37)	34 (33–37)	39 (38–40)	**<0.001**	0.153	**<0.001**	**<0.001**
Birth weight (grams)	2514 ± 799	2010 ± 634	3139 ± 482	**<0.001**	**<0.001**	**<0.001**	**<0.001**
Preterm birth (<37 week) (n, %)	37 (44.0%)	27 (79.4%)	15 (12.7%)	**<0.001**	**<0.001**	**<0.001**	**<0.001**
Cesarean section (n, %)	64 (76.2%)	28 (82.4)	69 (58.5%)	**0.005**	0.464	**0.009**	**0.011**
Fetal distress (n, %)	7 (8.3%)	8 (23.5%)	5 (4.2%)	**0.002**	**0.025**	0.225	**<0.001**
APGAR score at the first minute	9 (8–9)	8 (6–9)	9 (8–9)	**<0.001**	**0.005**	0.082	**<0.001**
APGAR score at the fifth minute	10 (9–10)	9 (8–10)	10 (10–10)	**<0.001**	**0.003**	**0.032**	**<0.001**
NICU admission (n, %)	27 (32.1%)	23 (67.6%)	13 (11%)	**<0.001**	**<0.001**	**<0.001**	**<0.001**
CANO (n, %)	45 (53.6%)	28 (82.4%)	20 (16.9%)	**<0.001**	**0.004**	**<0.001**	**<0.001**

Abbreviations: NICU: neonatal intensive care unit; CANO: composite adverse neonatal outcome.

## Data Availability

The data presented in this study are available on request from the corresponding author. The data are not publicly available due to privacy restrictions.
